# Research on Anti-Interference Performance of Spiking Neural Network Under Network Connection Damage

**DOI:** 10.3390/brainsci15030217

**Published:** 2025-02-20

**Authors:** Yongqiang Zhang, Haijie Pang, Jinlong Ma, Guilei Ma, Xiaoming Zhang, Menghua Man

**Affiliations:** 1 School of Communication Engineering, Hangzhou Dianzi University, Hangzhou 310018, China; yqz@hdu.edu.cn; 2 School of Information Science and Engineering, Hebei University of Science and Technology, Shijiazhuang 050018, China; phj@stu.hebust.edu.cn (H.P.); mjl@hebust.edu.cn (J.M.); 3 Shijiazhuang Campus, Army Engineering University of PLA, Shijiazhuang 050003, China; maguilei@aeu.edu.cn

**Keywords:** electromagnetic protection mode, spiking neural network, traditional artificial neural network, network connection damage, anti-interference performance

## Abstract

Background: With the development of artificial intelligence, memristors have become an ideal choice to optimize new neural network architectures and improve computing efficiency and energy efficiency due to their combination of storage and computing power. In this context, spiking neural networks show the ability to resist Gaussian noise, spike interference, and AC electric field interference by adjusting synaptic plasticity. The anti-interference ability to spike neural networks has become an important direction of electromagnetic protection bionics research. Methods: Therefore, this research constructs two types of spiking neural network models with LIF model as nodes: VGG-SNN and FCNN-SNN, and combines pruning algorithm to simulate network connection damage during the training process. By comparing and analyzing the millimeter wave radar human motion dataset and MNIST dataset with traditional artificial neural networks, the anti-interference performance of spiking neural networks and traditional artificial neural networks under the same probability of edge loss was deeply explored. Results: The experimental results show that on the millimeter wave radar human motion dataset, the accuracy of the spiking neural network decreased by 5.83% at a sparsity of 30%, while the accuracy of the artificial neural network decreased by 18.71%. On the MNIST dataset, the accuracy of the spiking neural network decreased by 3.91% at a sparsity of 30%, while the artificial neural network decreased by 10.13%. Conclusions: Therefore, under the same network connection damage conditions, spiking neural networks exhibit unique anti-interference performance advantages. The performance of spiking neural networks in information processing and pattern recognition is relatively more stable and outstanding. Further analysis reveals that factors such as network structure, encoding method, and learning algorithm have a significant impact on the anti-interference performance of both.

## 1. Introduction

With the rapid development of artificial intelligence technology, especially the wide application of large models such as ChatGPT-4 and DeepSeek, the problem of computing efficiency and energy consumption has become a hot topic of research. In this context, memristor, as a new type of electronic component with revolutionary potential, can realize data storage and computing power at the same time, and even simulate human brain neural networks. It provides innovative solutions to the problems of separation of storage and computing and high energy consumption in traditional computing architectures. The memristor can perform calculations directly in the storage unit, which greatly reduces the latency and energy consumption of data transmission. Due to its unique physical properties and similarity to biological neural networks, memristors are regarded as an ideal choice for building new neural network architectures [1]. With the rapid development of integrated circuits, artificial intelligence, the Internet of Things, and 5G communication technology, the electronic system is rapidly iterating in the direction of high integration, miniaturization, low power consumption, and intelligence [2]. Therefore, we want to minimize the number of memristors required to deploy the network through pruning technology to improve overall computational efficiency and energy efficiency. At present, various electromagnetic interferences have increasingly serious negative effects on electronic systems, which threaten the stability and security of networks [3]. In practical applications, electromagnetic interference can lead to the failure of memristor nodes or loss of connection, which can affect the performance and stability of the network. Therefore, when the network is disturbed or damaged, the anti-interference becomes the key factor for the normal operation of the network. Specifically, the anti-interference capability of the network refers to the ability of the network to maintain a certain degree of structural integrity and original functions after the nodes or connections are subjected to random or deliberate attacks. Therefore, the pruning algorithm is also used to simulate the damage of network connections, so as to explore the robustness of the network in the face of electromagnetic interference. The addition of pruning algorithms can not only help us to deeply understand the network’s performance in the face of interference, but also provide effective solutions to improve the reliability and efficiency of electronic systems. In this context, improving the anti-interference ability of the network not only has theoretical value but also lays a solid foundation for the reliability and stability of electronic systems in practical applications.

In recent years, Spiking Neural Network (SNN), as a novel neural network model, has attracted widespread attention. By using discrete spikes to transmit information, SNN is not only more in line with the information processing mode of the biological brain but also has lower energy consumption. These characteristics highlight the important application value of SNN in electromagnetism and promote the development and innovation of related technologies. SNN differs from traditional artificial neural networks, which are currently the most bio-interpretable Artificial Neural Network (ANN), whose nodes are spiking neuron models, and the edges are synaptic plasticity models [4,5]. Moreover, spiking neural networks use biologically plausible neuron models based on spike dynamics, whereas traditional artificial neural networks only use neurons based on static rates [6]. As a result, traditional ANNs tend to exhibit poor anti-interference to network impairments, which can easily lead to significant performance degradation or system failure. In contrast, SNN exhibits stronger anti-interference performance under network impairments due to their bio-inspired structure and dynamic adaptive mechanism. Guo et al. [7] constructed a spiking neural network with a small-world network and a stochastic network as its topology. They verified that the spiking neural network with small-world characteristics has excellent anti-interference function through electromagnetic interference simulation experiments. This also makes SNN an ideal choice for researching and developing the new generation of robust neuromorphic chips.

At present, there are relatively few studies on the effect of network connection impairments on the anti-interference performance of SNNs, and most of the existing research mainly focus on the impact of network topology on the anti-interference performance of SNNs. For example, Guo et al. [8] investigated the effect of topology on the damage resistance of SNNs, and the simulation results proved that SNNs with stronger biological rationality have better interference resistance. However, the simulation of the anti-interference performance of SNN under complex and changeable real network environments is not comprehensive enough, such as the research on network node damage or connection loss. Moreover, the comparative analysis between spiking neural networks and traditional artificial neural networks is still insufficient. Therefore, the anti-interference performance of SNN and ANN under the same degree of network connection damage is the focus of this paper. The anti-interference function of SNN and traditional ANN is studied under different degrees of network connection damage. Then, the SNN is compared with the traditional ANN, and the anti-interference mechanism of the network is further discussed.

The contributions of this paper are as follows:

(1) Verify the anti-interference performance of SNN and ANN. Construct two types of SNN models: VGG-SNN and FCNN-SNN, and compare them with traditional ANN (VGG and FCNN). VGG-SNN and FCNN-SNN are constructed to help us effectively and fairly evaluate the response ability of SNN and ANN in the face of interference and verify the anti-interference performance of SNN and ANN through experiments. The experimental results show that the SNN model has better anti-interference performance than the traditional ANN.
(2) Analyze the reasons why SNN has anti-interference performance. By analyzing the essential difference between SNN and ANN, the reasons for the different anti-interference performances of SNN and ANN are considered. The main difference between SNN and ANN is the encoding method.

## 2. Related Work

### 2.1. Artificial Neural Network

An artificial neural network is a network structure that is designed to handle practical problems with multiple nodes and output points [9]. Although the human brain and artificial neural networks are very powerful in information processing capabilities, there are still many differences between them. With the continuous advancement of artificial neural network technology, its applications have gradually expanded to various fields. AlphaGo’s victory over Go champion Lee Sedol in 2016 has attracted widespread attention to artificial neural networks. At present, artificial neural networks have spawned hundreds of models, among which MultiLayer Perceptron (MLP), Convolutional Neural Network (CNN), and Recursive Neural Network (RNN) are considered the most commonly used artificial neural network algorithms [10]. The structure of a basic ANN consists of three components: input layer, hidden layer, and output layer. This architecture is usually called a Fully Connected Neural Network (FCNN). Moreover, FCNN is a classic artificial neural network and is widely used to solve complex modeling problems [11]. However, compared to FCNN, CNN adopts a local connection approach, where each neuron is only connected to a portion of the neurons in the previous layer, rather than to all neurons [12]. Classic CNN models include LeNet-5 [13], AlexNet [14], VGGNet [15], GoogLeNet [16], and ResNet [17], all of which are improvements based on LeNet. The VGG network has been proven effective in many image-processing tasks due to its simple and moderately deep network structure. This study belongs to image classification tasks, so a deeper and more complex VGG model is chosen. In the VGG series, VGG11 has a relatively shallow network structure, which makes the model more efficient in training and inference processes. Additionally, due to the small number of layers in VGG11, this not only reduces computational complexity and memory consumption but also reduces the risk of overfitting. This is particularly advantageous when dealing with relatively limited datasets. Therefore, in order to adapt to the two datasets used in this research, VGG11 and FCNN are chosen as the experimental models representing ANNs, respectively.

### 2.2. Spiking Neural Network

The spike neuron model uses spike flow as the transmission form of data and these spike neurons are hierarchically connected to form a network, which is called a spiking neural network. In 1952, Hodgkin and Huxley studied the potential data of giant squid axons and proposed a detailed conductivity-based neuron model (H-H model) [18], successfully reproducing electrophysiological measurement results. However, the H-H model is limited in practical tasks due to its high computational complexity. Therefore, subsequent low-order neuron models usually simplify the H-H model to reduce computational costs. Currently, several common first-order neural models include the Integrate-and-Fire (IF) model [19] and the Leaky Integrate-and-Fire (LIF) model [20]. The IF model provides a good compromise between biological plausibility and computational cost. However, this model mainly focuses on the temporal dynamics of neurons and considers the spatial structure and spatial information processing less. The LIF model summarizes the information transmission process of neurons into three modes: charging, discharging, and resetting, and the model can enhance the spatial perception ability of neurons through appropriate parameter settings and network structure design [21]. Therefore, in this paper, the LIF model was adopted as the SNN element based on spike and simulation. Moreover, to fairly compare the anti-interference performance between SNN and ANN, this research constructs two SNN models with network structures similar to VGG and FCNN, named VGG-SNN and FCNN-SNN, respectively.

### 2.3. Comparison of Advantages and Disadvantages Between ANN and SNN

After years of development, ANN technology has been widely used for various tasks, and its tools and frameworks have matured. It adopts a backpropagation algorithm, which is easy to train and suitable for large-scale datasets. The ANN particularly excels in processing static data such as image and text recognition. However, ANN is inferior to SNN in processing time-series data and dynamic signals, and ANN has higher resource and energy consumption in large-scale networks, which differs significantly from the operating principles of biological nervous systems. SNN simulates the spike-firing mechanism of the biological nervous system, which has higher biological interpretability and significant energy-saving advantages. Its event-driven calculation method reduces energy consumption compared to traditional ANN. SNN is particularly suitable for processing time series data and dynamic signals, which can effectively capture temporal features and demonstrate stronger anti-interference ability in the face of noise and interference [22]. However, the training algorithm for SNN is relatively complex and existing training methods such as backpropagation are difficult to apply. In addition, the development tools and frameworks of SNN are not yet mature and there are few applications. The network structure and parameter settings of SNN are also complex and require more professional knowledge. In practical applications, algorithms often face various noises and interferences. Studying their anti-interference performance can help improve the stability and reliability of algorithms in complex environments. The advantages and disadvantages of ANN and SNN make them play unique roles in different scenarios. By studying their anti-interference performance, suitable network models can be selected for different application scenarios, thereby improving the overall robustness of the system.

### 2.4. Pruning Algorithm

Neural networks use pruning algorithms to remove redundant parts in the network, such as connections, neurons, and filters, to reduce the size of the network. This method is widely regarded as an effective optimization strategy [23]. It reduces the resource requirements of the network, including memory, computing, and energy consumption [24]. In recent years, some groundbreaking studies have explored neural network pruning algorithms. Pruning operations are widely used in the fields of ANN and SNN. In the field of ANN, Xue et al. [25] proposed an automatic filter pruning algorithm called AFPruner based on the average similarity of feature maps and reverse search genetic algorithm (RSGA). It automatically searches for the optimal combination of pruning proportions for all convolution layers, evaluates the filter similarity through the feature map average similarity, and then trims the similarity filter. Wang et al. [26] proposed a network channel pruning scheme based on sparse learning and genetic algorithms to achieve a better balance between pruning ratio and accuracy. Bellec et al. [27] further proposed Deep Rewriting (Deep R) as a pruning algorithm for ANN and Long Short-Term Memory (LSTM) spiking neural networks. Deep R has the ability to achieve target connections without further training. Afterward, Liu et al. [28] applied Deep R to the prototype chip of the second-generation SpiNNaker system. Deep R achieved a classification accuracy of 96.6% on an extremely sparse network, which verified the effectiveness of the Deep R algorithm on memory-limited hardware. Ebid et al. [29] proposed a correlation-based pruning algorithm that systematically reduces the network size by identifying and deleting redundant neurons based on their activation correlates. The core idea of the algorithm is to selectively trim neurons while compensating their contributions to the network, so as to maintain the fidelity of the model on different datasets. In the field of SNN, some people attempt to mimic the way the human brain learns to enhance SNN pruning. Among them, Deng et al. [30] combined STBP and ADMM to compress the SNN model in two aspects: connection pruning and weight quantization, greatly reducing memory space and baseline operations. Kappel et al. [31] proposed synaptic sampling to optimize network construction and weights by modeling the spinal motion of spike neurons as Bayesian learning. Chen et al. [32] proposed the Gradient Re routing Pruning Algorithm (Grad R), which redefines the gradient as a new synaptic parameter and better explores the network structure by fully utilizing the competition between connection pruning and regeneration. In addition, Chen et al. also applied the Deep R pruning algorithm to SNN. However, there is a lack of systematic studies that directly compare the reduction in accuracy after SNN and ANN pruning. Therefore, in order to fill this research gap, this paper conducted a pruned direct comparison of SNN and ANN on the same task and dataset. Since Deep R can be effectively applied to SNN and ANN, it proves the universality and flexibility of the Deep R pruning algorithm. Therefore, the Deep R pruning algorithm is chosen to be built into the four network models used in this paper.

### 2.5. Research on Anti-Interference of Spiking Neural Network

In recent years, multiple papers have studied the anti-interference performance of spiking neural networks. Guo et al. [33] studied the influence of small-world model parameters on the anti-interference function of neural networks. The experimental results showed that spiking neural networks with higher clustering coefficients and lower average path lengths have better anti-interference functions. Subsequently, Guo et al. [34] constructed two complex spiking neural network models (CSNN) and studied the influence of topology on the damage resistance of CSNN. The experimental results show that the robustness of the topology structure is consistent with the anti-damage function of CSNNs, indicating that the anti-damage ability of both types of CSNNs is affected by the topology structure. In further research, Guo et al. [8] constructed five types of spiking neural network models and compared their anti-interference capabilities. The experimental results show that under Gaussian white noise stimulation, complex SNN is superior to small-world SNN, small-world SNN is superior to scale-free SNN, and these are superior to random SNN and conventional SNN. This indicates that SNN with more biological rationality has better anti-interference ability. However, the above research mainly focuses on the impact of network topology on anti-interference performance and there is relatively little research on the impact of SNN anti-interference performance under network connection damage. Therefore, this research focuses on the anti-interference performance of SNN under network connection damage.

## 3. Methods

The objective of this research is to investigate the anti-interference performance of SNN and ANN under the same degree of network connection damage. Therefore, two kinds of spiking neural network models, VGG-SNN and FCNN-SNN, were designed in this research. These two models are similar to the network structure of VGG and FCNN, respectively, and both use the LIF model as the basic neuron model for constructing SNN. In addition, the Deep R pruning algorithm was applied to the training process of ANN and SNN. By calculating the standard deviation of the weights in the weight matrix, all activity weights with absolute values less than or equal to the set value were pruned to simulate network connection damage. This method helps evaluate the anti-interference performance of ANN and SNN under network connection damage.

### 3.1. Spike Neuron Model

Simple spiking neuron models have provided many insights into neural coding, memory, network dynamics, and deep learning. The LIF neural model has found the best balance between biological rationality and practicality and is the most commonly used neural model for simulating large-scale spiking neural networks. Therefore, the LIF model was chosen as the basic neural model. The LIF model, as a compromise between the complex dynamic characteristics of biological neurons and simple mathematical forms, can be described using differential functions [35,36]:

(1) $$\tau \frac{dV(t)}{dt}=-V(t)+X(t),$$

where τ is the time constant, $V\left(t\right)$ is the membrane potential of the postsynaptic neuron at time t, and $X\left(t\right)$ is the integrated current input by the presynaptic neuron at time t. For the convenience of computer simulation, it is necessary to discretize and recursively solve the differential formula to obtain a simple iterative representation of the LIF-SNN layer for inference and training. The specific calculation formula is as follows:

(2) $$U^{t,n}=H^{t-1,n}+X^{t,n},$$

(3) $$S^{t,n}=Hea(U^{t,n}+u_{th}),$$

(4) $$H^{t,n}=V_{reset}S^{t,n}+(\beta U^{t,n})⨀(1-S^{t,n}),$$

where t and n represent time steps and layers, and $U^{t,\;n}$ represents the membrane potential generated by coupling, which is generated by the coupling of spatial features $X^{t,\;n}$ and time inputs $H^{t-1,\;n}$. $u_{th}$ determines the output spike tensor, and $S^{t,\;n}$ is a given or zero threshold. $Hea\left(\cdot \right)$ is a Heaviside step function, which satisfies $Hea\left(x\right)=1$ when *x* ≥ 0, otherwise $Hea\left(x\right)=0$. $V_{reset}$ is the reset potential set after activating the output spike, β represents the decay factor, and ⊙ represents the multiplication of elements.

### 3.2. Structure of Spiking Neural Network Model

In this research, the SpikingJelly framework is used to construct two types of SNN models, which are designed and evaluated for different datasets. The first model is named VGG-SNN, which has a network structure similar to the VGG model. This model is specifically designed to process the human action dataset used in this paper, which is named the Radar action dataset. The second model is named FCNN-SNN, which has a network structure similar to FCNN and is used to process the MNIST dataset. By constructing an SNN model with a similar structure to ANN, the performance difference between SNN and traditional ANN can be compared more directly. Therefore, the two SNN models proposed are intended to help this study effectively and fairly evaluate the response-ability of SNN and ANN in the face of interference and verify the immunity performance of SNN and ANN through experiments.

#### 3.2.1. VGG-SNN

**Figure 1:**
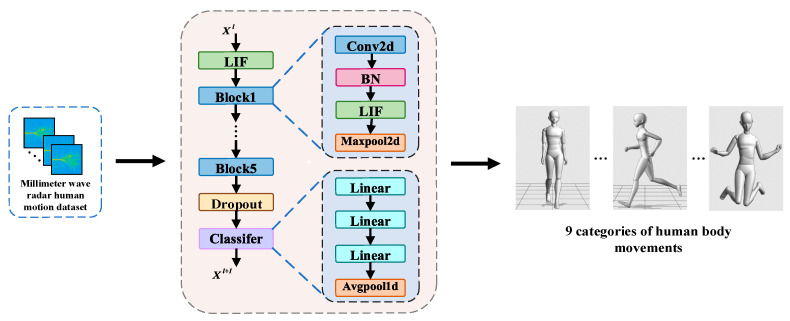
Network structure diagram of VGG-SNN model.

The structure of the VGG-SNN model is shown in Figure 1, which consists of neuron layers that generate spikes and synaptic layers including convolutional layers, pooling layers, and fully connected layers. The synaptic layer defines the connection pattern of synapses between spike neurons, which is used for spatially correlated feature extraction or classification. The structure of this model is similar to VGG11, which not only extracts spatial features through the convolutional layer and the pooling layer but also enhances the processing ability of dynamic changes through the temporal encoding mechanism of spike neurons.

In the input layer, the input feature map is defined as X, $X\in R^{C\times H\times W}$, C representing the number of input channels, H and W representing the height and width of the feature map. Firstly, perform a 2D convolution operation on the input feature map and the calculation formula is as follows:

(5) $$Y(i,j)=\sum_{c=0}^{C-1}\sum_{m=0}^{k-1}\sum_{n=0}^{k-1}W(c,m,n)\cdot X(c,i+m,j+n)+b,$$

where Y(i,j) represents the value of the output feature map Y at position (i,j), W(c,m,n) represents the weight value at position (m,n) in the c channel of the convolution kernel W. m and n are the sizes of the convolution kernel, and i and j are the position indices of the output feature map. Next, perform batch normalization (BN) on the output feature map Y using the following formula:

(6) $$Z(i,j)=\gamma \cdot \frac{Y(i,j)-\mu }{\sqrt{\sigma^{2}+\varepsilon }}+\beta ,$$

where Z(i,j) is the standardized output obtained after BN operation, μ and $\sigma^{2}$ are the mean and variance of the input feature map. γ and β are learnable scaling and offset parameters, and ε is a very small constant used to prevent the denominator from being zero.

Next, the feature map Z(i,j) that has undergone the above operations is input into the LIF model for further encoding, where the probability of each neuron firing a spike is proportional to the intensity of the corresponding pixel in the input feature map Z(i,j). The input current is generated by multiplying the input features with synaptic weights and this current accumulates in the membrane potential of subsequent neurons, as shown in Formula (1). Whenever the membrane potential of a posterior neuron exceeds its discharge threshold, the neuron produces an output spike and resets the membrane potential. Otherwise, the membrane potential decays exponentially over time. Each layer of neurons (excluding the output layer) executes the process sequentially based on the weighted peaks received from the previous layer. The formula for calculating the total sum of weighted peak sequences passed from the previous layer over time is as follows:

(7) $$x_{i}^{l-1}(t)=\sum_{t}\sum_{k}x_{i}^{l-1}(t-t_{k})\cdot e^{-\beta (t-t_{k})},$$

(8) $$I_{j}^{l}(t)=\sum_{i=1}^{n^{l-1}}\left(w_{ij}^{l-1}x_{i}^{l-1}(t)\right),$$

where $x_{i}^{l-1}\left(t\right)$ represents the sum of spike sequences of the i-th pre neuron in layer l−1 over time, t and $t_{k}$ represent the current time and the previous time, $t_{k}$ ≤ t. $x_{i}^{l-1}\left(t-t_{k}\right)$ represents the spike that occurred in the first neuron of layer l−1 from moment t to moment $t_{k}$, and $e^{-\beta \left(t-t_{k}\right)}$ represents the time decay factor, which gradually reduces the influence of past time points over time. $I_{j}^{l}\left(t\right)$ represents the integrated current that accumulates to the membrane potential of the j-th posterior neuron in layer l at time t. $n^{l-1}$ is the number of pre-neurons in layer l−1, and $W_{ij}^{l-1}$ represents the synaptic weight from the i-th neuron to the j-th neuron in layer l−1. Therefore, according to the above formula, the calculation formula for the membrane potential update process is as follows:

(9) $$V_{t}=V_{t-1}+\frac{1}{\tau }(I_{t}-V_{t-1}),$$

where $I_{t}$ is the integrated current input to the presynaptic neuron at moment t. $V_{t-1}$ is the membrane potential of the t−1 neuron at the previous moment, and $V_{t}$ is the membrane potential of the neuron at the current moment.

In the final classification layer, the firing threshold of the neurons is set very high, which prevents these output neurons from producing spiking outputs. In the last layer, the weighted input signals of each neuron accumulate continuously in this layer and these accumulated membrane potentials gradually decay over time to simulate the membrane potential dynamics of biological neurons. In the last time step, the accumulated membrane potential is evaluated to obtain a stable and representative output distribution. When the membrane potential reaches a preset high threshold, the neurons produce spikes. Finally, the cumulative spike count in time step T is divided by the total number of time steps T, which is obtained as the peak frequency for each neuron. This quantified spike frequency distribution is the final network output for classification decisions. The process is shown in Formula (10):

(10) $$output=\frac{X_{mem}^{l}(T)}{T}\;,$$

where, $X_{mem}^{l}$ refers to the cumulative spike count in time step T. Finally, the output features are trained. Each potential connection of the neural network can be active or dormant at any time during training. Therefore, the Deep R pruning algorithm is applied to the training process of SNN and ANN as an optimizer to simulate network connection damage.

Specifically, the pruning process determines whether to retain each synaptic connection by evaluating its importance. In the implementation process, this study first trains the network to obtain the initial connection weights. Then, the Deep R pruning algorithm is used to evaluate the connection weights to determine which connections can be cut. The feature of the Deep R pruning algorithm is to ensure that the maximum demand for network connections during training will not exceed a certain limit.

First, the connection matrix is initialized. To control the sparsity of the network, the sparsity p is set to represent the probability of each connection being activated. When initializing the connection matrix, each connection k generates a random number $r_{k}$ using a uniform distribution. If the generated random number is less than p, the connection is activated (value 1), otherwise, it is set to zero (value 0). This means that every connection has an equal chance of being set to zero, which is to ensure randomness and diversity in the network. This ensures that only a portion of the connections in the connection matrix are active, thus achieving sparsity. By adjusting the sparsity, the percentage of pruning can be randomly set. This setting can be adjusted based on the dataset, application-specific accuracy requirements, and power/delay budgets for a more flexible and efficient pruning strategy. The formula for calculating sparsity is as follows:

(11) $$c_{k}=\left\{\begin{array}{c} 1,\;\;\;if\;r_{k}<p\\ 0,\;\;\;if\;r_{k}\geq p \end{array}\right.,$$

where, $c_{k}$ is an element in the connection matrix, 1 indicates active connection and 0 indicates dormant connection. Dormant connections no longer participate in weight updates, which reduces network connectivity.

**Figure 2:**
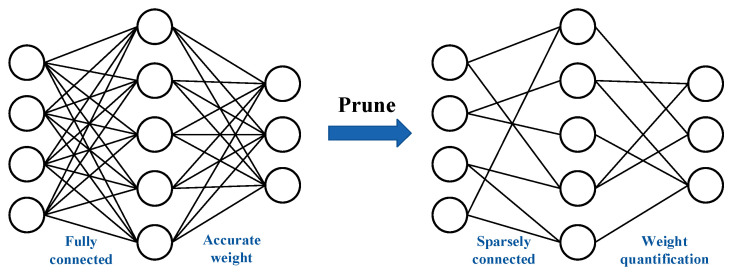
Network pruning process.

Next, the parameters are initialized. The Deep R pruning algorithm assigns a connection parameter $\theta_{k}$ and a constant $s_{k}\in \left\{-1,\;1\right\}$ to each connection k. When $\theta_{k}<0$, the synaptic connection is set to sleep, the contact connection is removed from the network and no longer considered for renewal, with a corresponding weight of $w_{k}$ = 0. When $\theta_{k}\geq 0$, the synaptic connection is active and the corresponding network weight is $w_{k}=s_{k}\theta_{k}$. The connection parameters are represented in such a way that each connection encodes not only information about whether it is active but also the weight of the connection. This method can eliminate unnecessary connections and thus simplify the network structure. The pruning process is shown in Figure 2.

Then, the gradient update of the parameters is carried out. Only those connections that have not been cut and whose weight absolute value has not fallen to 0 will have their parameters updated with the gradient. The error function used in this algorithm is cross entropy error. The update rule includes a $L_{1}$ regularization term and introduces the noise term $\sqrt{2\eta T}v_{k}$, where $v_{k}$ is the noise sampled from a Gaussian distribution with zero mean and unit variance. The introduction of noise makes the parameter update random, which combines the characteristics of gradient descent and random walk. The formula for gradient updating is shown in Formula (12).

(12) $$\theta_{k}=\left\{\begin{array}{cc} \theta_{k}-\eta \frac{\alpha }{\partial \theta_{k}}E_{X,Y^{*}}(\theta )-\eta \alpha +\sqrt{2T\eta v_{k}}, & if\;c_{k}=1\\ \theta_{k}, & if\;c_{k}=0 \end{array}\right.,$$

where, $T=\eta \frac{\sigma^{2}}{2}$ is the temperature parameter, which is used to adjust the flatness of the posterior distribution. When T=1, the posterior distribution is restored. When T>1, the posterior distribution is flattened. When T<1, the posterior distribution is sharpened. η represents the learning rate, which controls the magnitude of each update. α represents the regularization coefficient, which controls the strength of the regularization term.

Finally, the reconnection strategy is implemented. This study determines whether to activate a new connection or delete an existing connection based on the state of the current parameters and connectivity constraints. This process randomly selects dormant connections and activates them according to a set policy. The rules are as follows: If the connection parameter $\theta_{k}<0$, the connection is set to sleep and does not participate in updates. To ensure that K active connections are always maintained during training, new connections are randomly selected from the dormant connections and activated. Therefore, the training objectives of the algorithm are as follows:

(13) $$p^{*}(\theta )=\left\{\begin{array}{l} 0\;\;\;\;if\;violates\;the\;constraint\\ \frac{1}{K}p^{*}{\left(\theta \left|X,Y^{*}\right.\right)}^{\frac{1}{H}}\;otherwise \end{array}\right.,$$

where K is a normalization constant and X and Y represent the input and output data, respectively. The pseudo-code of the Deep R pruning algorithm is shown below (Algorithm 1) [24], which is a summary of the above content.
**Algorithm 1:** Deep R Algorithm**Input**: $i,N_{i},k,\theta_{k},\eta$ **Output**: $\theta_{k\prime }$ 1: **for** *i* in [1, $N_{i}$] **do** 2:  **for** all active connections $k\left(\theta_{k}\geq 0\right)$ **do** 3:   $\theta_{k}\leftarrow \theta_{k}-\eta \frac{\partial }{\partial \theta_{k}}E_{X,\;Y^{*}}\left(\theta \right)-\eta \alpha +\sqrt{2\eta H}v_{k}$; 4:   **if** $\theta_{k}<0$ **then** set connection *k* dormant; 5:  **end** 6:  **while** number of active connections lower than *K* **do** 7:    select a dormant connection k′ with uniform probability and activate it; 8:    $\theta_{k\prime }\leftarrow 0$ 9:  **end** 10: **end**

#### 3.2.2. FCNN-SNN

**Figure 3:**
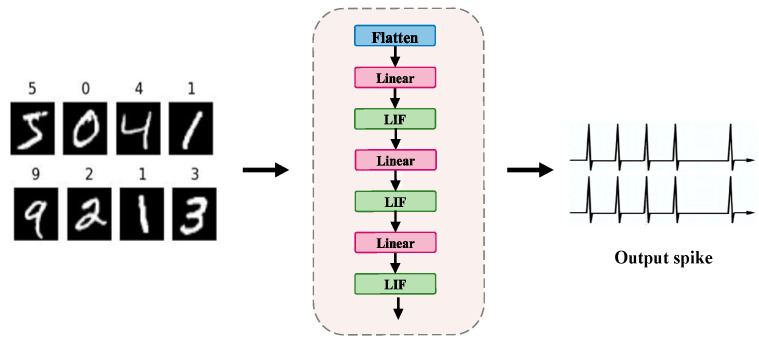
Network structure diagram of FCNN-SNN model.

The constructed FCNN-SNN model is a fully connected neural network defined by the number of input neurons *n*, the number of output neurons *m*, and an *m × n* weight matrix. The number of input neurons *n* can be adjusted according to preprocessing but by default, it is 784, corresponding to the grayscale pixels in each training sample. In this paper, the output layer is set to consist of 500 neurons and is used to classify 10 categories of the MNIST dataset. The MNIST dataset contains 60,000 training images and 10,000 test images. The fully connected network structure of the FCNN-SNN model is shown in Figure 3.

Firstly, the input multidimensional data x is flattened into a one-dimensional vector for subsequent layer processing. The flattened data is then passed through the linear layer, as shown in the following formula:

(14) $$x_{flat}=reshape(x,(N,784)),$$

(15) $$h_{l}=W_{l}\cdot x_{flat},$$

where N represents the number of samples, $W_{l}$ is the weight matrix of the l-th layer, and $h_{l}$ represents the output of the l-th layer. After passing through the fully connected layer, the output is passed to the LIF layer, which converts the feature map into a spike sequence of Poisson distribution. The process is shown in Formulas (7)–(10) in Section 3.2.1. The pruning operation during the training process is detailed in Section 3.2.1.

## 4. Results

In this section, the anti-interference performance of two constructed SNN models and two traditional ANN models was evaluated on the Radar action dataset and the MNIST [14] dataset.

### 4.1. Datasets

#### 4.1.1. Radar Action Dataset

The Radar action dataset was constructed by our team. During the collection process, special attention is paid to the diversity of the collection personnel, including factors such as gender, height, and body shape. The dataset collected motion samples from three experimental participants, with 330 samples collected for each type of human motion, totaling 2970 sample data. When designing human motion, it is necessary to select representative samples that cover different categories of motion and have universality. In order to improve the practicality and adaptability of sports design, the laboratory also considered the naturalness and fluency of sports. Therefore, a total of 9 types of human movements were designed, including forward walking, backward walking, left walking, right walking, jumping, forward running, backward running, left running, and right running.

**Figure 4:**
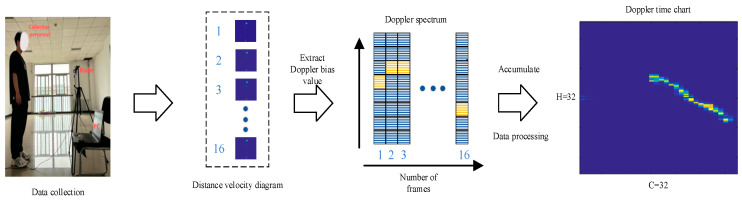
Radar action dataset collection and processing process. The yellow color in the figure represents higher Doppler frequencies, while the blue part represents lower Doppler frequencies.

Next, the processing process of the radar signal is explained and the acquired radar signal is processed by 2D-FFT twice to obtain a frame of a “range-velocity diagram”. This two-dimensional image can fully reflect the distance and velocity characteristics of the target. In order to remove static target interference, the frame difference method is used for preprocessing. The specific method is to accumulate several frames to calculate the background frame and then subtract the background frame from the original image to obtain the de-noised range-velocity graph. This pre-processing step effectively improves the clarity of the signal and lays the foundation for subsequent analysis. The data collection and processing process is shown in Figure 4, and the calculation formula is as follows:

(16) $$X_{bk}=\frac{1}{s}\sum_{k=1}^{s}\sum_{i=1}^{m}\sum_{j=1}^{n}x_{k}^{\left(i.j\right)},$$

(17) $$X_{bk}^{\prime }(x_{k}^{\left(r,d\right)})=\left\{\begin{array}{ll} \sum_{r=1}^{n}\left[\sum_{d=1}^{\frac{m}{2}}x_{bk}^{\left(r,d\right)}+\sum_{d=\frac{m}{2}+2}^{m}x_{bk}^{\left(r,d\right)}\right], & d\neq \frac{m}{2}+1\\ \sum_{r=1}^{n}x_{bk}^{\left(r,d\right)}, & d=\frac{m}{2}+1 \end{array}\right.$$

where $X_{bk}$ is the background frame, s represents the cumulative number of background frames in the distance velocity map, m and n are the total number of pixels in the distance axis and velocity axis of the distance velocity map, respectively.

When the human body performs different actions in front of the millimeter wave radar, the frequency of the radar echo signal will change. Based on this Doppler frequency shift feature, this research constructs a Doppler-time graph as input. By using the Doppler shift of human motion, the Doppler spectrum describing the speed of each frame is extracted from the distance-time graph and accumulated over time. Specifically, multiple frames of radar data are accumulated to calculate the Doppler spectrum, which is stacked by time to build a Doppler-time graph. This two-dimensional image not only reflects the dynamic characteristics of the target but also is one of the commonly used inputs in human motion recognition.

The performance of deep learning algorithms largely depends on the quality of the sample data. In order to improve the availability of samples, the paper normalizes the obtained Doppler-time graph sample base. First, standardize the image size, set it to 32 × 32 and adjust all samples to the same resolution. After that, the pixel values are normalized and the grayscale is mapped to the [0, 1] range. Finally, the data were cleaned to eliminate invalid and mislabeled samples, and the human movement dataset of this paper was successfully constructed. These normalized processing steps help to eliminate differences between images, which improves model generalization and improves overall algorithm performance.

#### 4.1.2. MNIST Dataset

The MNIST dataset contains 70,000 handwritten digit images, 60,000 training images, and 10,000 test images. The training set consists of digits handwritten by 250 different people, half from high school students and half from staff members, and the test set followed the same proportions. The MNIST dataset ensures that the training set and the test set are collected by different collectors. Each image is a 28 × 28 pixel point and the dataset will be stored by converting the data from one image into a 784 one-dimensional vector.

### 4.2. Experimental Setup

In the experiment, SpikingJelly 0.0.4 [37] and Pytorch 2.1 frameworks were used to design and evaluate SNNs. By setting different sparsity parameters, simulate different degrees of network connection damage, to reflect real-world situations where some neurons or connections may fail due to environmental interference or network attacks. Adjusting sparsity can help researchers observe the robustness and recovery ability of the network under different degrees of damage. When there is no need to simulate network damage, both SNN and ANN use Adam as the optimizer. The Adam optimizer adaptively adjusts the learning rate of each parameter according to the first and second moment estimates of the gradient.

To verify the anti-interference performance of SNN in different environments, the VGG-SNN model was used to process the Radar action dataset constructed in the laboratory. Due to the rich temporal information and complex dynamic features contained in this dataset, as well as various environmental noises, it is suitable for evaluating the anti-interference performance of the model under dynamic changes and noise interference. The VGG-SNN model incorporates a deep convolutional neural network structure, which can effectively extract complex time-domain features from time series data. Therefore, VGG-SNN and VGG models are suitable for processing Radar action datasets, which can better verify and evaluate their anti-interference and adaptability in practical applications. In addition, a shallow FCNN-SNN model is used for the MNIST dataset. The MNIST is a relatively simple and well-structured dataset with low computational complexity. Therefore, using the FCNN-SNN model to process the MNIST dataset can reduce computational complexity and complete training faster with higher accuracy.

Table 1 summarizes the hyper-parameter settings of the SNN model on different datasets and provides a reference for subsequent experiments. These hyper-parameter settings help researchers better understand the network’s performance under various conditions, ensuring the reliability and reproducibility of experimental results.

### 4.3. Experimental Results and Analysis

In many practical applications, especially in scenarios involving network connection damage, data often suffers from class imbalance. Simply using accuracy as an evaluation metric may lead to bias towards the main categories, as even if the model’s predictions for a few categories are poor, the overall accuracy may remain at a high level. Therefore, accuracy and recall can more comprehensively reflect the model performance in different categories, especially in recognizing minority categories. The core of studying anti-interference performance lies in understanding the model performance in the face of noise and interference. By using precision, recall, and F1-score, the model performance under uncertain conditions can be better evaluated.

Therefore, to fairly compare the anti-interference performance of the SNN and ANN, five indicators including sparsity, accuracy, precision, recall, and F1-score were comprehensively considered. In the experiment, five sparsity values were set to simulate varying degrees of network connection damage, namely 30%, 40%, 50%, 60%, and 70%, and used as control groups for ablation experiments. By comparing and analyzing the performance differences between the SNN model and ANN model on two datasets under the same damage conditions, a comprehensive evaluation of the SNN’s anti-interference capability can be conducted. The percentage decrease in accuracy in Table 2, Table 3, Table 4 and Table 5 shows the average value, float (top), and minimum value (bottom), while the other data shows the average value, float (top), and maximum value (bottom).

#### 4.3.1. Comparison of Experimental Results Between VGG-SNN and VGG

In this section, in order to more intuitively demonstrate the performance differences, Table 2 and Table 3 only show the experimental results with sparsities of 30% and 70%. These two tables respectively reflect the accuracy changes of VGG-SNN and VGG under the same damage conditions on the Radar action dataset. According to the data in Table 2 and Table 3, when the sparsity is 30%, the accuracy of VGG-SNN decreases by 5.83%, while the accuracy of VGG decreases by 18.71%. When the sparsity is 70%, the accuracy decrease rate of VGG-SNN is 15.95%, while the decrease rate of VGG increases to 27.24%. From this, it can be seen that under lower damage conditions, the accuracy decrease in VGG-SNN is significantly smaller than that of VGG, which means that VGG-SNN can more effectively maintain its performance.

**Figure 5:**
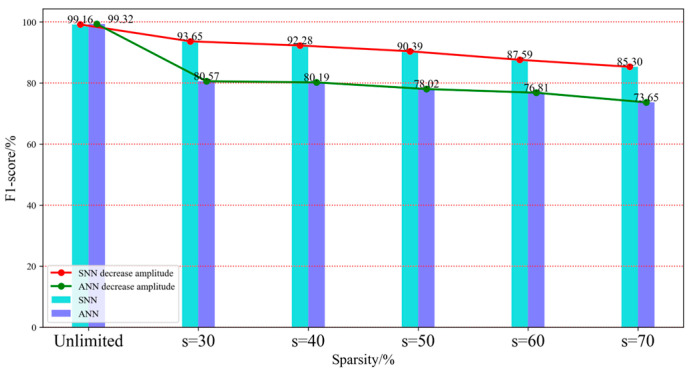
F1-score histogram of VGG-SNN and VGG on Radar action dataset.

In addition, Figure 5 shows the comparison results of VGG-SNN and VGG on the F1-score index under five sparsity levels. F1-score, as a harmonic mean of precision and recall, can effectively comprehensively consider the accuracy and completeness of the model. This indicator can clearly identify whether SNN can maintain its predictive ability and to what extent it can resist external interference in the event of network damage. From Figure 5, it can be seen that in the Radar action dataset, VGG-SNN exhibits higher a F1-score, indicating that its performance in both accuracy and recall is superior to VGG. This means that VGG-SNN can not only more accurately identify positive samples, but also effectively reduce false alarm rates and improve overall performance.

The Radar action dataset contains rich temporal data that can reflect dynamic changes in human body movements and may involve various environmental factors and noise interference. The above experimental results demonstrate the significant advantages of SNN in dynamic information processing under network connection damage conditions. Especially under high noise and damage conditions, SNN exhibits stronger robustness and adaptability, and can better cope with complex practical application scenarios.

#### 4.3.2. Comparison of Experimental Results Between FCNN-SNN and FCNN

Similar to Section 4.3.1, Table 4 and Table 5 only show the experimental results with sparsities of 30% and 70%. These two tables show the experimental results for FCNN-SNN and FCNN, respectively. When the sparsity is 30%, the accuracy of SNN decreases by 3.91%, while the accuracy of FCNN decreases by 10.13%. When the sparsity is 70%, the accuracy decrease rate of FCNN-SNN is 8.23%, while the decrease rate of FCNN increases to 28.17%. The MNIST dataset is static image data of handwritten digits, focusing on image recognition and feature extraction. In this relatively simple task, the lower decrease exhibited by FCNN-SNN not only demonstrates its effectiveness in maintaining recognition ability but also emphasizes its robustness in processing static images.

**Figure 6:**
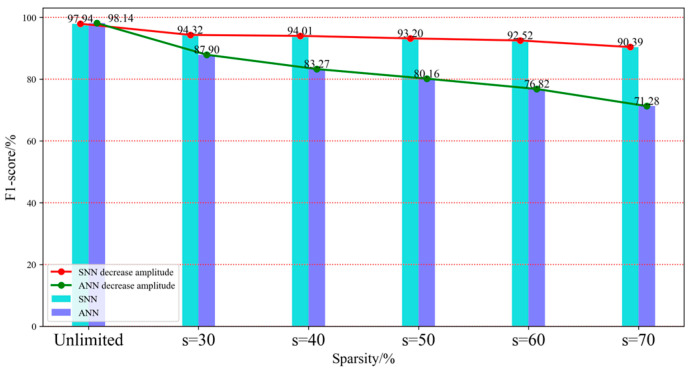
F1-score histogram of FCNN-SNN and FCNN on MNIST dataset.

Figure 6 shows the comparison results of FCNN-SNN and FCNN on the F1-score index under five sparsity levels. In the MNIST dataset, FCNN-SNN is similar to the trend of the Radar action dataset and also shows better anti-interference ability than FCNN. FCNN-SNN demonstrates stronger robustness in handling handwritten digit recognition tasks and can maintain high classification accuracy in incomplete or disturbed data.

Therefore, the aforementioned findings indicate that in scenarios of network connection damage, SNN can more effectively capture critical features and process input information accurately even when partial connections fail. In contrast, the ANN decreases significantly under the same conditions. The above reflects the vulnerability of ANN when the network connection is damaged, which may lead to the loss of key information.

In summary, by comparing the anti-interference performance of SNN and ANN on the Radar action dataset and MNIST dataset, this research can demonstrate that SNN exhibits higher anti-interference performance and adaptability in the face of network connection damage. Whether in millimeter wave radar data or handwritten digit images, the accuracy decrease in SNN is significantly smaller than that of ANN. This shows that SNN can more effectively maintain its performance and demonstrate its strong anti-interference performance. With the increase in sparsity, that is, the aggravation of network connection damage, although the accuracy decline of SNN has also increased, its performance is still better than ANN. This phenomenon further validates the potential of SNN in handling dynamic and uncertain environments, especially in application scenarios that require high reliability, such as handwritten digit recognition and real-time monitoring systems. These results indicate that SNN exhibits stronger anti-interference ability and classification performance in the face of network damage.

#### 4.3.3. Resource Analysis of SNN and ANN

In this section, the resource usage of SNN and ANN is analyzed statistically considering the resource constraints in practical applications. The purpose is to understand the resource usage characteristics of SNN and ANN, providing an important basis for the effective deployment of deep learning models in resource-constrained environments. The memory and training time data in Table 6 are averages. In Table 6, it can be observed that after the Deep R pruning algorithm is added, the number of parameters, memory usage, and training time of both SNN and ANN are significantly reduced. This shows that the pruning algorithm plays an important role in optimizing model structure and resource utilization, which enables the two models to run with less computing resources and memory usage while maintaining relative performance. This optimization is especially important for model deployments in real-world applications, as they are able to operate efficiently in resource-constrained environments.

Although the training time of SNN is usually greater than that of ANN, this is mainly due to the complex dynamic mechanism used by SNN to process information, which leads to high computational complexity. This time difference suggests that when choosing a model, researchers must carefully weigh the training time against the desired accuracy. It is worth noting that the accuracy of SNN decreases relatively little after adding pruning, which indicates that SNN can still maintain high performance in a highly sparse network structure. This makes SNN advantageous in applications requiring high accuracy, such as time series analysis and biological signal processing. In contrast, although ANN’s training time is shorter, the decline in accuracy may be greater after pruning. Therefore, the choice of SNN or ANN should be based on the specific application needs, especially to find the right balance between accuracy and training efficiency.

From the perspective of resources, SNN and ANN have their advantages and disadvantages. SNN performs well in terms of parameter count, memory usage, and power consumption, which makes it suitable for use in resource-constrained environments. ANN has advantages in computational efficiency and training time, which is suitable for dealing with large-scale data and complex tasks. This study considers that it is a very interesting and promising direction to explore the integration of SNN characteristics into ANN. This combination can help make up for the shortcomings of ANN in processing time series data and dynamic signals. For example, the event-driven mechanism and time coding characteristics of SNN can enhance the sensitivity of ANN to timing information, which makes it more robust in complex environments. Similarly, integrating the characteristics of ANN into SNN can significantly improve the training efficiency of SNN, such as an efficient backpropagation algorithm and powerful training capability. This complementary feature not only reduces the training time of SNNS but also improves their performance on large datasets.

In summary, SNN achieves a good balance between the number of parameters, memory usage, training time, and accuracy, which demonstrates the effectiveness of pruning techniques. By incorporating pruning algorithms, SNN can significantly reduce resource requirements while maintaining high accuracy. By introducing pruning algorithms, SNN is able to significantly reduce resource requirements while maintaining high accuracy, which makes SNN particularly suitable for applications in resource-constrained environments. In addition, pruning algorithms facilitate the use of SNN in fast iterative and efficient deployment scenarios, such as real-time data processing and intelligent monitoring systems. Therefore, future research can further explore the application of SNN in edge computing and discuss its integration scheme with edge intelligent devices. In addition, SNN can be combined with the distributed architecture of edge computing to enable more efficient data processing and decision-making, which will drive the development of smart cities, the Internet of Things, and industrial automation. Through these studies, SNN is expected to play a greater role in edge computing, which provides strong support for the realization of intelligence and automation.

## 5. Discussion

In this chapter, the reasons for the different anti-interference performances of SNN and ANN are analyzed. The main reason is that SNN and ANN are encoded differently. SNN uses spike coding information, which is transmitted by the frequency and time of the spike. In this encoding mode, information is transmitted and expressed through the neuron’s spike release time, which allows information to be transmitted in a more flexible way [34]. The information is encoded through the frequency and time of the spike, which makes SNN more robust to small connection damage or input noise. Even if some connections are lost, the remaining spikes can still transmit information. In contrast, ANN is usually encoded numerically, which relies on continuous values for information transfer and outputs a node-based activation function. Loss of connections can result in significant changes in activation values, which can affect overall performance. When a connection is lost, the activation value in the ANN can change significantly, which can lead to a disruption in the transmission of information and seriously affect overall performance. In the case of a high-noise environment or unstable connection, the robustness of ANN is poor, and it is often difficult to maintain stable output.

In order to verify the above views, this study tries to transfer the weight of SNN to ANN. The aim is to better understand how differences in information encoding between SNNS and ANN affect their robustness, and whether the characteristics of SNNS can effectively enhance the performance of ANN. Table 7 shows the detailed data of the experimental results:

According to the above results, we observed that when the weight of SNN was transferred to ANN without pruning, the accuracy of ANN was slightly improved. In addition, after the pruning algorithm is applied, the weight of SNN is transferred to the pruned ANN, and the accuracy of the ANN is slightly higher than the original value. However, the difference in accuracy caused by weight transfer is within the range of statistical deviation, which is far less significant than the difference between pruned ANN and pruned SNN. The experimental results show that the performance advantage of SNN after pruning is not due to weight transfer but may be related to other factors. Therefore, our preliminary guess is that the excellent accuracy of the pruned SNN is due to the characteristics of its spike coding information. The sparsity of the spike code not only gives SNN high efficiency in processing information but also enables it to capture and retain critical information more accurately, while effectively reducing redundant calculations. This finding provides important clues for further research into the unique advantages of SNN. In future studies, it is necessary to delve deeper into the mechanism of SNN’s operation, in particular how spike coding affects its performance and how this advantage can be better applied to practical tasks.

## 6. Conclusions

In this paper, two spiking neural network models, VGG-SNN and FCNN-SNN, are first constructed and systematically compared with the classical VGG model and FCNN model. During the model construction process, the Deep R pruning algorithm is introduced to simulate network connection damage and this combination can dynamically adjust the network structure. The redundant parameters are reduced by pruning so that the performance of different networks after connection damage is more effectively evaluated. Next, the anti-interference performance of the above two SNNs is compared and analyzed with that of the two traditional ANNs under the same degree of network connectivity damage level. The experimental results show that SNNs exhibit stronger perturbation resistance when faced with the same degree of network connectivity damage. This phenomenon suggests that the spike coding and information processing of SNN enables it to retain important information more effectively during information transfer and feature extraction, thus significantly improving the robustness of the model. Finally, the reasons why SNN is better than traditional ANN are considered, analyzed, and verified by experiments. Specifically, SNN may be due to its flexible spike coding method, which makes the SNN model maintain high recognition accuracy in complex environments.

In conclusion, this research not only validates the potential of SNN in image recognition tasks but also reveals its advantages in anti-interference performance. This feature gives SNN a unique advantage in edge computing environments, which facilitates fast, intelligent data processing and real-time feedback. In the future, this paper proposes to explore further the application range of SNN, especially its integration into edge computing. The application of SNN enables smart devices to handle complex tasks without relying on cloud computing, especially in the field of the Internet of Things and autonomous driving. Therefore, the application of SNN in future intelligent systems will not be limited to image recognition, but will also extend to a variety of real-time decision-making and control tasks, which will push smart devices to be more efficient and more autonomous.

## Figures and Tables

**Table 1:** Default values of hyper-parameters for SNN models on different datasets.

Hyper-Parameters	Radar Action	MNIST
Max Epoch	200	200
Batch Size	128	128
Learning Rate	0.001	0.001
T	8	20
$V_{th}$	1.0	1.0
τ	2.0	2.0

**Table 2:** Experimental results of VGG-SNN on Radar action dataset.

Model	Sparsity	Acc (%)	Percentage Decrease in Accuracy (%)	Precision (%)	Recall (%)	F1-Score (%)
VGG-SNN	--	98.93 ± 0.12% 99.16	--	98.91 ± 0.16 99.18	99.00 ± 0.10 99.15	98.79 ± 0.21% 99.16
VGG-SNN + Deep R	30%	91.96 ± 1.11% 93.37	4.48 ± 0.66% 4.79	93.00 ± 0.98% 94.13	91.82 ± 1.25% 93.37	91.44 ± 1.53% 93.65
VGG-SNN + Deep R	70%	81.59 ± 1.34% 83.33	15.84 ± 0.81% 14.73	84.91 ± 1.89% 87.10	81.42 ± 1.61% 83.33	82.19 ± 2.05% 85.3

**Table 3:** Experimental results of VGG on Radar action dataset.

Model	Sparsity	Acc (%)	Percentage Decrease in Accuracy (%)	Precision (%)	Recall (%)	F1-Score (%)
VGG	--	97.85 ± 1.32% 99.32%	--	97.42 ± 0.13% 97.99	96.99 ± 0.12% 97.96	97.2 ± 0.28% 97.98
VGG + Deep R	30%	80.34 ± 1.03% 82.62	16.46 ± 0.97% 15.81%	80.65 ± 0.64% 81.67	78.97 ± 0.92% 79.62	78.67 ± 1.21% 80.57
VGG + Deep R	70%	72.74 ± 2.29% 75.26	25.23 ± 4.61 22.06%	72.37 ± 1.88% 74.78	68.94 ± 2.12% 72.49	70.69 ± 1.37% 73.65

**Table 4:** Experimental results of FCNN-SNN on MNIST dataset.

Model	Sparsity	Acc (%)	Percentage Decrease in Accuracy (%)	Precision (%)	Recall (%)	F1-Score (%)
FCNN-SNN	--	98.11 ± 0.10% 98.25	--	97.73 ± 0.15% 97.95	97.36 ± 0.36% 97.94	97.68 ± 0.13% 97.94
FCNN-SNN + Deep R	30%	92.99 ± 1.01% 94.40	4.96 ± 0.85% 3.92%	92.38 ± 1.38% 94.33	92.57 ± 1.29% 94.31	93.16 ± 0.78% 94.32
FCNN-SNN + Deep R	70%	87.57 ± 2.12% 90.16	9.76 ± 2.34% 6.7%	88.67 ± 1.73% 90.62	88.53 ± 1.12% 90.16	87.88 ± 1.96% 90.39

**Table 5:** Experimental results of FCNN on MNIST dataset.

Model	Sparsity	Acc (%)	Percentage Decrease in Accuracy (%)	Precision (%)	Recall (%)	F1-Score (%)
FCNN	--	97.78 ± 0.53% 98.60%	--	97.55 ± 0.12% 97.65	97.08 ± 0.34% 97.89	97.45 ± 0.10% 98.14
FCNN + Deep R	30%	86.10 ± 1.31% 88.53	10.43 ± 1.64% 9.16%	86.38 ± 1.13% 88.05	86.26 ± 1.26% 87.97	86.65 ± 1.02% 87.90
FCNN + Deep R	70%	69.38 ± 2.21% 72.15	28.9 ± 1.82% 26.38%	67.94 ± 1.98% 70.19	68.52 ± 1.41% 70.66	68.39 ± 2.17% 71.28

**Table 6:** Comparison of resource indicators between SNN and ANN.

Index	FCNN-SNN	FCNN-SNN + 0.7 Deep R	FCNN	FCNN + 0.7 Deep R
Parameter quantity	535,040	374,073	535,818	374,528
Internal memory	1027 MiB	327 MiB	1039 MiB	875 MiB
Training time	17.03 s/epoch	14.82 s/epoch	14.21 s/epoch	11.76 s/epoch
Acc	98.11 ± 0.10% 98.25%	87.57 ± 2.12% 90.16%	97.78 ± 0.53% 98.60%	69.38 ± 2.21% 72.15%

**Table 7:** The ANN performance comparison table with SNN weights is introduced.

Model	Whether to Add Pruning	Accuracy of the Original Weight (%)	Accuracy of Introducing SNN Weights (%)
FCNN	×	97.78 ± 0.53% 98.60%	98.12 ± 0.78% 99.03%
	√	86.10 ± 1.31% 88.53%	87.62 ± 1.64% 89.61%

## Data Availability

The MNIST dataset utilized in this study is publicly accessible. To access it, you have the option to use tools such as PyTorch for downloading purposes. Alternatively, you can opt for manual downloads directly from the following website: http://yann.lecun.com/exdb/mnist/ (MNIST accessed on 17 February 2025). The Radar action dataset provided in this study can be provided at the request of the corresponding author due to privacy concerns and the lack of a dedicated open data repository.
